# Dual Valorization of Lignin as a Versatile and Renewable Matrix for Enzyme Immobilization and (Flow) Bioprocess Engineering

**DOI:** 10.1002/cssc.202100926

**Published:** 2021-07-05

**Authors:** Ana I. Benítez‐Mateos, Stefania Bertella, Jean Behaghel de Bueren, Jeremy S. Luterbacher, Francesca Paradisi

**Affiliations:** ^1^ Department of Chemistry Biochemistry and Pharmaceutical Sciences University of Bern Freiestrasse 3 3012 Bern Switzerland; ^2^ Laboratory of Sustainable and Catalytic Processing Institute of Chemical Sciences and Engineering École Polytechnique Fédérale de Lausanne (EPFL) 1015 Lausanne Switzerland

**Keywords:** biocatalysis, enzyme immobilization, flow chemistry, lignin, renewable resources

## Abstract

Lignin has emerged as an attractive alternative in the search for more eco‐friendly and less costly materials for enzyme immobilization. In this work, the terephthalic aldehyde‐stabilization of lignin is carried out during its extraction to develop a series of functionalized lignins with a range of reactive groups (epoxy, amine, aldehyde, metal chelates). This expands the immobilization to a pool of enzymes (carboxylase, dehydrogenase, transaminase) by different binding chemistries, affording immobilization yields of 64–100 %. As a proof of concept, a ω‐transaminase reversibly immobilized on polyethyleneimine‐lignin is integrated in a packed‐bed reactor. The stability of the immobilized biocatalyst is tested in continuous‐flow deamination reactions and maintains the same conversion for 100 cycles. These results outperform previous stability tests carried out with the enzyme covalently immobilized on methacrylic resins, with the advantage that the reversibility of the immobilized enzyme allows recycling and reuse of lignin beyond the enzyme inactivation. Additionally, an in‐line system also based on lignin is added into the downstream process to separate the reaction products by catch‐and‐release. These results demonstrate a fully closed‐loop sustainable flow‐biocatalytic system based exclusively on lignin.

## Graphical abstract

**Lignin is applied** as a renewable support for enzyme immobilization. A range of lignins with different functionalization is developed to expand immobilization to different enzymes. The integration of enzyme‐lignin derivatives in flow reactors shows higher biocatalyst stability than with typical material supports. Lignin is doubly valorized through use as a product purification scavenger in flow.

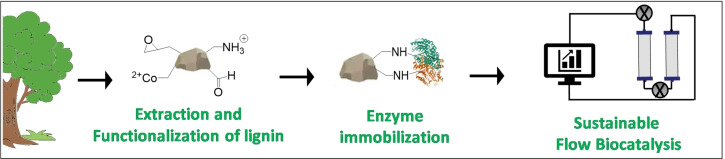

## Introduction

As society searches for more eco‐friendly technologies, both science and industry are increasingly focusing on the beneficial properties of lignin, a major component of lignocellulosic biomass, which has been traditionally relegated to being an unutilized by‐product.^[1]^ Its bioavailability, biodegradability and non‐toxicity makes lignin a sustainable and cost‐efficient material compared to fossil‐based products.^[2]^ However, the valorization of lignin is hampered by traditional lignocellulosic biomass fractionation processes which typically separate its three major components (cellulose, hemicellulose, and lignin).^[3]^ Specifically, the harsh conditions generally associated with these processes typically lead to random recondensation of the lignin making it difficult to breakdown or exploit given its resulting disordered structure.^[4]^ Despite this, during the last decades, lignin has found several uses. It has been used in agriculture as a fertilizer and in animal feed,^[5]^ in the construction industry as emulsifying and dispersing agent,^[6]^ and in the food industry^[1]^ among others. Additional and more advanced uses of lignin in high‐value applications are also appearing. For instance, the field of bioplastic manufacturing has incorporated lignin derivatives in its materials. Lignin has also been used as a starting material for the production of bioactive molecules.^[7]^ Additionally, fabrication of nanoparticles, electrochemical nano‐composites, and production of advanced biofuels are a set of examples of the innovative uses of lignin during the last years.^[8]^

Recently, the combination of lignin with other components resulting in novel hybrid materials has also been reported.[^9^, ^10^, ^11^] A common theme in all of these applications is that they have been able to work around the fact that the lignin that was employed had a disordered and difficult‐to‐characterize structure, which has ultimately limited lignin's use in many applications.

Recently, a new fractionation strategy was reported that used aldehyde as protecting groups to stabilize the lignin β‐O‐4 bonds through formation of stable acetals, which prevents this recondensation and preserves a native‐like lignin structure.[^12^, ^13^] Specifically, this process allows to obtain an uncondensed lignin that can be easily converted to small aromatic units in near‐theoretical yields and an additional oligomeric and aromatic fraction after catalytic hydrogenolysis. Furthermore, this aldehyde‐assisted fractionation can be further exploited using multifunctional aldehydes to simultaneously extract and functionalize the lignin with non‐native functional groups^[12]^ Such functionalization strategies open new opportunities for exploiting lignin in unique ways.^[14]^

In the field of biocatalysis, a sustainable approach to produce high‐value chemicals, efforts for the valorization of lignin have focused mainly on ligninolytic enzymes. Many laccases, peroxidases, and even tyrosinases are intensively studied not only for their role in the degradation of lignin but also as effective catalysts for pharmaceutical production, textile industry and wastewater treatment.^[15]^ In contrast, less attention has been paid to lignin as a useful material itself for other applications in biocatalysis, for example as a matrix for enzyme immobilization. There is yet no universal support that suits all enzymes and applications, but a broad scope of materials is available, such as synthetic polymers, acrylic resins and active membranes.[^16^, ^17^] Even though the stability of the immobilized enzyme is frequently good, the high cost and the potential toxicity of some supports are the main drawbacks of the current enzyme immobilization supports. The advantageous properties of lignin turn this organic polymer into an attractive but poorly explored alternative for immobilization of enzymes. Hitherto, only a few examples of lignin derivatives have been used as supports to immobilize enzymes,[^9^, ^18^, ^19^, ^20^, ^21^] mainly relying on unselective hydrophobic interactions between the enzyme and the polymer. Such a strategy cannot be used for most of the enzymes whose hydrophobic residues are buried within the structure and its distortion could lead to catalyst deactivation. In parallel, the integration of immobilized enzymes in continuous flow reactors is steadily growing due to the increased efficiency, sustainability, and process automation.[^22^, ^23^] However, the variety of matrixes that have been integrated in either (bio)flow reactors or downstream processes (e. g., product separation) is limited to commercially available materials. In this context, developing a more selective and universal method for immobilizing enzymes could greatly expand the use of this sustainable substrate in biocatalysis.

In this work, we harness the aldehyde‐stabilization method for extraction and simultaneous functionalization of lignin to develop a series of functionalized lignin with controlled reactive groups (e. g., aldehyde, amino, epoxy, metal chelate, monosaccharide) greatly expanding enzyme immobilization possibilities. We both characterized these immobilized biocatalysts, and integrated enzymes immobilized on lignin in a flow reactor for the continuous production of valuable chemicals. Additionally, lignin was used as a scavenger material for downstream processes in flow, resulting in a fully sustainable closed‐loop biosynthetic process.

## Results and Discussion

**Figure 1:**
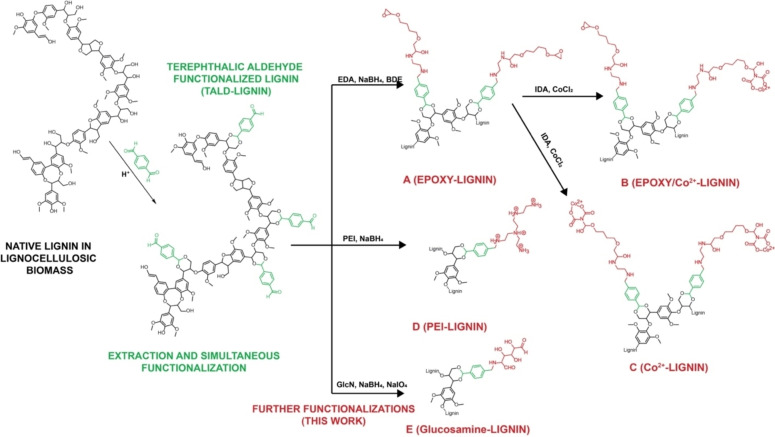
Extraction and simultaneous functionalization of lignin with terephthalic aldehyde (TALD‐lignin) and further functionalization shown in this work: A) Epoxy‐lignin; B) epoxy/Co^2+^‐lignin; C) Co^2+^‐lignin; D) PEI‐lignin; E) glucosamine‐lignin.

Following a recently developed protocol for the simultaneous extraction and functionalization of lignin with terephthalic aldehyde (TALD),^[14]^ we performed fractionation of lignocellulosic biomass in presence of an increasing concentration of TALD to extract lignins functionalized with 1.5 mmol g^−1^ of aldehyde groups (Figure 1 and Figure S1 in the Supporting Information). This TALD‐lignin was then used for further functionalization and enzyme immobilization.

The TALD‐lignin was firstly activated with epoxy groups (129 μmol g^−1^) to exploit the enzyme immobilization through the nucleophile groups on the protein surface (e. g., lysine, cysteine, tyrosine) as typically done with other material supports.^[24]^ Due to the poor reactivity of the epoxy groups, a second functional group is frequently added to the support to attract the enzyme to the surface of the support and further react covalently with the enzyme. Following this premise, we added cobalt chelate groups, creating a heterofunctional support where 45 μmol g^−1^ of epoxy supports remained unaltered (see the Supporting Information). Moreover, a monofunctional lignin with only cobalt chelate groups was developed for the reversible immobilization of His‐tagged proteins. To expand the binding chemistries between the enzyme and the lignin surface, the TALD‐lignin was covered with amino groups which create a positively charged surface for the ionic immobilization of enzymes. Finally, a more hydrophilic aldehyde‐activated lignin was developed by adding glucosamine (Figure S2).

### Enzyme immobilization on differently functionalized lignin matrices

Taking advantage of the aldehyde functionalities on the lignin surface, the immobilization of two different enzymes, lysine‐6‐dehydrogenase from *Geobacillus stearothermophilus* (Gs‐Lys6DH)^[25]^ and alcohol dehydrogenase from *Geobacillus stearothermophilus* (Bs‐ADH),^[26]^ was initially studied through the generation of imine bonds followed by reduction. This covalent immobilization strategy usually affords a high stability to the immobilized enzyme on traditional supports, although the recovered activity after immobilization is decreased (Table 1).^[27]^ To improve the recovered enzyme activity and to expand the immobilization on lignin to other enzymes, the TALD‐lignin was functionalized with several different groups: amino (PEI), epoxy, cobalt chelates, and both epoxy and cobalt chelates (Figures 1 and S2). The different functionalities allowed the immobilization of Gs‐Lys6DH, Bs‐ADH and other industrially relevant enzymes from *Halomonas elongata*, ω‐Transaminase (HeWT) and Pyrrolidine‐5‐carboxylate (He−P5C),[^28^, ^29^] using several irreversible and reversible chemical bonds (Figure S3), reaching 64–100 % of immobilization yield. Despite the low recovered activity (<1–17 %) compared to traditional immobilization supports,[^29^, ^30^, ^31^] a positive trend was detected when shifting from the irreversible (on aldehyde and epoxy groups) to the reversible binding chemistries (on PEI and cobalt chelate groups; Table 1). This effect is well known when immobilizing enzymes on other typical supports such as agarose or acrylic microbeads.^[32]^

**Table 1:** Immobilization parameters of enzymes on lignin derivatives.^[a]^

Enzyme	Reactive group	Immobilization chemistry	Immobilization yield^[b]^ [%]	Recovered activity^[c]^ [%]	Immobilized activity on the lignin^[d]^ [U g^−1^]
Gs‐Lys6DH	aldehyde	Covalent bond	100	3	0.1
	Cobalt chelate	Affinity interaction	94	7	0.16
	PEI	Ionic interaction	98	10	0.2
Bs‐ADH	aldehyde	Covalent bond	64	<1	<0.1
	Cobalt chelate	Affinity interaction	70	5	0.13
HeWT	Epoxy	Covalent bond	92	8	0.8
	Epoxy and cobalt chelate	Covalent bond driven by affinity interaction	95	9	0.9
	Cobalt chelate	Affinity interaction	95	14	1.4
	PEI	Ionic interaction	100	17	1.8
He−P5C	Cobalt chelate	Affinity interaction	95	<1	<0.1
	PEI	Ionic interaction	98	<1	<0.1

[a] 5 mg of protein were used with 1 g of support in all cases. The specific activity of the purified free enzymes was: Gs‐Lys6DH 0.5 U mg^−1^, Bs‐ADH 0.7 U mg^−1^, HeWT 2.1 U mg^−1^, He−P5C 5.7 U mg^−1^. [b] Immobilization yield=[(activity of the free enzyme ‐ activity of the supernatant after immobilization)/activity of the free enzyme]×100. [c] Recovered activity=(specific activity of the free enzyme/specific activity of the immobilized enzyme)×100. [d] Immobilized activity on the lignin=activity of the immobilized enzyme per gram of lignin.

To better understand the enzyme immobilization on lignin, the enzymes were labeled with a fluorophore (FITC) for their visualization under confocal fluorescence microscopy. Gs‐Lys6DH on TALD‐lignin was studied as an example of irreversible binding, while HeWT on PEI‐lignin was selected as a reversible case. Even at low magnification (objective 10x), a uniform distribution of Gs‐Lys6DH was observed not only among the different particles but also across the inner section of each particle, likely due to the homogeneous distribution of aldehyde groups and the longer incubation time (3 h) with the enzyme. Contrastingly, HeWT was non‐uniformly distributed and mainly located on the outer surface of the particles (Figure S4A). This effect might be the result of a faster incubation with the enzyme (1 h) that promotes only the ionic adsorption of the enzyme on the outer surface of the PEI‐lignin. These results are in agreement with previously reported studies where enzymes immobilized predominantly on the exposed surface of the support (as opposed to those that diffuse through the porous matrix) display a higher activity due to better mass transfer ability from the bulk to the catalyst.[^33^, ^34^, ^35^] However, more sophisticated studies would be needed to decipher the exact effect observed. Nevertheless, the particle size of the TALD‐lignin is very similar to the acrylic microbeads as observed by microscopy, which facilitates comparison with previous work (Figure S4B).

We hypothesized that the homogeneous enzyme distribution and associated lower recovered activity may be an effect of the hydrophobic interactions between the aromatic structure of the lignin and the hydrophobic residues of the enzyme, as described for other immobilized biocatalysts.^[35]^ To test this, the aldehyde groups of lignin were reduced to avoid covalent binding of the Gs‐Lys6DH (Figure S5), and the enzyme was then left in contact with the support which resulted in more than 90 % of immobilization yield, clearly showing a strong hydrophobic affinity. The interactions between the enzyme and the lignin were shown to be reversible by SDS‐PAGE analysis (Figure S5). In addition, the enzyme was immobilized on lignin with different levels of aldehyde functionalization (0.44, 1.51, and 1.89 mmol g^−1^). In all cases, more than 99 % of immobilization yield was achieved. However, higher aldehyde contents (1.51 and 1.89 mmol g^−1^) resulted in 30‐fold improvements in recovered activity compared to the lowest loading (0.44 mmol g^−1^) for Gs‐Lys6DH on TALD‐lignin (Figure S6). These results suggest that the hydrophobic interactions may decrease when the aldehyde content is increased and consequently the recovered activity of the immobilized enzyme is higher. In the case of HeWT immobilized on PEI‐lignin, the activity following immobilization was 3‐fold higher. In this case, the support is coated with a highly hydrophilic cationic polymer (PEI) which may diminish the hydrophobic interactions resulting in an enhanced recovered activity of the immobilized enzyme in all cases. Due to these results, the lignin with 1.5 mmol g^−1^ was employed for further experiments.

To avoid undesired hydrophobic interactions during enzyme immobilization, several additives (ethanol, SDS, glycerol, triton) were trialed during the immobilization process.[^35^, ^36^] Nevertheless, no significant improvements over the resulting activity (U g^−1^) of the biocatalyst were detected (Figure S7). Additionally, lignin was functionalized with glucosamine to increase the hydrophilicity of the support following a modified protocol reported before for functionalization of acrylic resins.^[37]^ Then, the Gs‐Lys6DH was immobilized on glucosamine‐lignin yielding a 2‐fold increase on the recovered activity compared to the enzyme immobilized on TALD‐lignin (Figure S8). Overall, however, this approach did not outperform the PEI‐lignin strategy.

It was noteworthy that the hydrophobic interactions were advantageous for the immobilization on monofunctional epoxy‐lignin. The epoxy groups require longer incubation times with respect to aldehydes and often the catalyst does not reach full immobilization. For this reason, another anchoring point (i. e., metal chelate, primary amine) is typically added to the support.^[38]^ In this case, the hydrophobicity of lignin acts as a driving force and the enzyme immobilization happened in a short time yielding 92 % immobilization (Table 1).

### Characterization of immobilized HeWT on PEI‐lignin: reusability and stability

**Figure 2:**
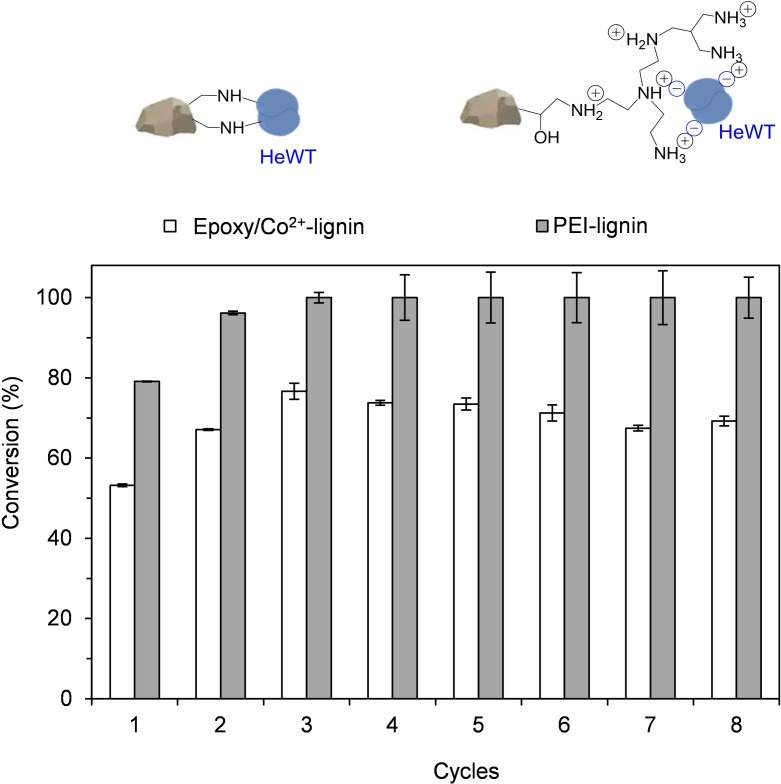
Recycling and reuse of HeWT immobilized (5 mg g^−1^) on Epoxy/Co^2+^‐lignin and PEI‐lignin. Top: Schematic representation of the immobilized biocatalysts. Bottom: Graph depicting the operational stability. Each reaction cycle corresponds to 2 h at 37 °C. The reaction mixture contained 2.5 mm pyruvate, 2.5 mm *S*‐MBA, and 0.1 mm PLP in 10 mm phosphate buffer at pH 8.

Transaminases are attractive catalysts from an industrial perspective and they have been extensively exploited.[^39^, ^40^] The immobilized HeWT was therefore selected for further study. The reusability of two differently immobilized HeWT (epoxy/Co^2+^‐lignin and PEI‐lignin), was tested and compared. In the first case the enzyme was covalently bound to the lignin, while in the second case HeWT was reversibly immobilized via ionic interactions (Figure 2). In both cases, the operational stability of the biocatalyst was maintained for at least 8 consecutive batch reactions (Figure 2). However, the PEI‐lignin‐HeWT reached a 30 % higher conversion than the epoxy/Co^2+^‐lignin‐HeWT due to higher activity (U g^−1^) on the matrix (Table 1). Importantly, despite the ‘weak’ ionic interactions between HeWT and the PEI‐lignin, the enzyme remained attached to the support after 8 batch cycles (Figure S9A). To decipher whether these results were also caused by other interactions between the enzyme and the support, the immobilized biocatalyst was incubated with a salt solution (3 M NaCl) to disrupt the ionic interactions, but some enzyme was still found to be bound to the resin (Figure S9B), likely through hydrophobic forces. This effect was confirmed by SDS‐PAGE after additional incubation of the immobilized biocatalyst with ethanol, leading to full removal of the enzyme from the PEI‐lignin (Figure S9B). Both reversible interactions (ionic and hydrophobic) between HeWT and PEI‐lignin are an advantage for exploiting lignin as a sustainable matrix for enzyme immobilization because the support can be recycled to immobilize fresh enzyme following inactivation of the preceding enzyme over time (Figure S9B).

To maximize the catalytic efficiency of HeWT immobilized on PEI‐lignin, we optimized the protein loading of HeWT on the support, obtaining 5 mg g^−1^ (1.8 U mg^−1^) as the most convenient option (Figure S10). Furthermore, we tested the stability of the PEI‐lignin‐HeWT with different cosolvents and pH values. In agreement with prior results of the immobilized HeWT on acrylic resins,^[30]^ the immobilized enzyme outperforms its free counterpart in presence of 20 % of solvent (isopropanol, acetonitrile, ethanol, ethyl acetate, dimethyl sulfoxide; Figure S11A). Then, the stability of the biocatalyst was tested following incubation at pH 6–12 for 30 min. The residual activity of the immobilized enzyme was comparable to the free enzyme at pH 8–10 (Figure S11B). Nevertheless, the stabilizing effect of the immobilization on PEI‐lignin was highlighted at the most extreme pH values tested (6–7 and 11–12).

### Enzyme and cofactor co‐immobilization on PEI‐lignin

Transaminases are cofactor‐dependent and therefore the addition of exogenous cofactor (PLP in this case) is often needed, thus increasing the process costs. As an alternative and inspired by a previously described methodology to co‐immobilize the PLP (pyridoxal‐5’‐phosphate),[^41^, ^42^] we harnessed the PEI coating of the lignin to co‐immobilize HeWT with PLP (Figure S12A and S12B). The cofactor was co‐immobilized by reversible interactions which allow it to travel from the catalytic site of the enzyme to the polymeric bed of PEI without diffusing out the lignin environment. PLP immobilization yielded 7 μmol g^−1^ as reported before for other support materials.^[41]^ Interestingly, the operational stability of the co‐immobilized biocatalyst equaled the immobilized biocatalyst fed with exogenous cofactor, avoiding the external addition of cofactor in each reaction cycle (Figure S12C). These results support the potential of lignin for use as matrix not only for enzyme immobilization but also for cofactor immobilization when needed.

### Integration of HeWT immobilized on PEI‐lignin in a flow reactor

**Figure 3:**
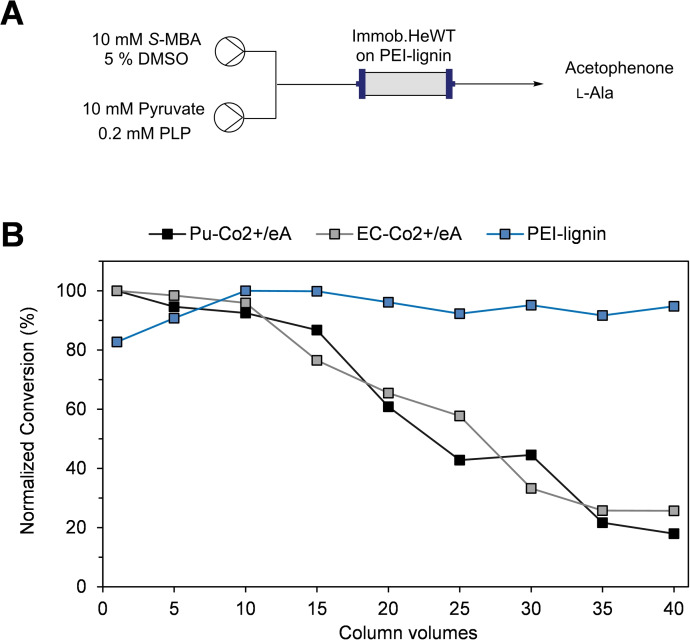
Stability testing of reversibly immobilized HeWT (1 mg g^−1^) on different supports: A) Scheme representation of the flow system. B) Comparison of HeWT stability when immobilized on Pu‐Co^2+/^eA, EC‐Co^2+^/eA, and PEI‐lignin. The full normalized conversion relates to 10 mm acetophenone for Pu‐Co^2+^/eA and EC‐Co^2+^/eA, and 5 mm for PEI‐lignin. Each column volume corresponds to 5 min. Temperature: 37 °C. Flow‐rate: 0.2–0.3 mL min^−1^. The results of HeWT immobilized on Pu‐Co^2+/^eA and EC‐Co^2+^/eA were extracted from ref. [41].

Flow biocatalysis has become a powerful tool to scale‐up synthetic reactions.^[23]^ To evaluate whether lignin is a suitable support for integration into flow systems, the PEI‐lignin‐HeWT was packed into a packed‐bed reactor (PBR; Figure 3A). The stability of the biocatalyst was tested for a model deamination reaction achieving a constant conversion rate of 60 % for 40 column volumes. Owing to the reversible nature of the immobilization chemistry, such stability was unexpected and in fact it significantly outperformed previously tested reversible preparations on the acrylic resins Pu‐Co^2+^/eA (Purolite ECR8215F activated with cobalt chelates and ethanolamine) and EC‐Co^2+^/eA (Resindion EC‐EP/S activated with cobalt chelates and ethanolamine).^[41]^ As reported before, HeWT immobilized on Pu‐Co^2+^/eA and EC‐Co^2+^/eA failed the stability test since the enzyme was partially eluted from the support, leading to 75–83 % drop‐off on conversion from 100 % in the first column volume to 17–25 % m.c. after 40 column volumes (Figure 3B). In contrast, HeWT immobilized on PEI‐lignin remained completely attached to the support after 40 cycles as observed by SDS‐PAGE (Figure S13). To compare the stability results of the three flow bioreactors, the normalized conversion is depicted in Figure 3B. Notably, the STY (space‐time yield) after 40 column volumes was improved by a factor of 2–3 when using HeWT immobilized on PEI‐lignin (8.15 g/L/h), while maintaining similar productivity and turnover numbers to those obtained when using the acrylic resins as material supports (Table S1).

In addition, the operational stability of the immobilized biocatalyst was analyzed under more challenging conditions. First, the flow reaction was performed without free cofactor added (only with natural bound PLP). The conversion rate was stable for 50 column volumes and just 10 % lower than the same biocatalyst where free cofactor was added continuously (Figure S14), in agreement with the operational stability of HeWT immobilized covalently on acrylic resins.^[41]^ Then, we studied the effect of the flow rate which is one of the key parameters for the productivity in flow systems. The flow rate of the reaction was modulated according to the retention time (*t*_R_) that was shortened from 5 minutes to 1 min without exogenous PLP added. The operational stability was maintained for 100 column volumes with a 18 % reduction on the conversion with respect to the flow bioreactor operating at 5 min (Figure S15A). Moreover, under these flow conditions (*t*_R_=1 min, no exogenous PLP) the STY was increased up to 35 g/L/h with a productivity of 37.7 mg_product_ mg_enzyme_^−1^ h^−1^. In terms of stability, HeWT reversibly immobilized on PEI‐lignin was equivalent to the biocatalyst when immobilized covalently on epoxy‐resin (Figure S15B).

**Figure 4:**
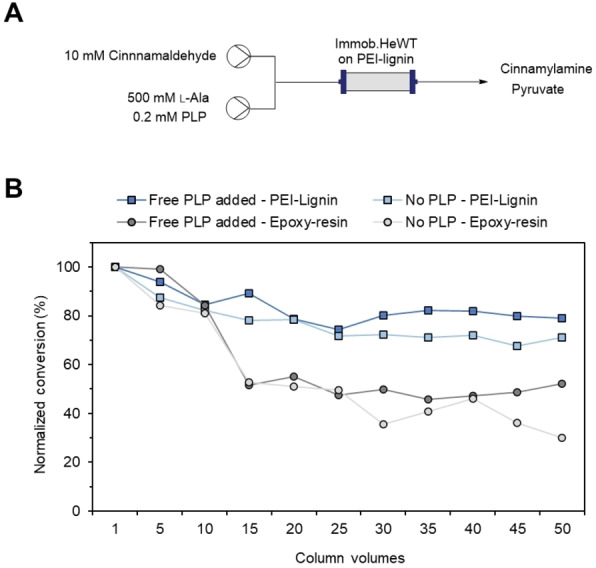
Continuous‐flow synthesis of cinnamylamine by immobilized HeWT. A) Schematic representation of the general set up for the flow biocatalytic reaction. B) Stability of HeWT immobilized on different supports. Each column volume corresponds to 2 min. Temperature: 37 °C. Flow‐rate: 0.35 mL min^−1^. The results of the biocatalyst immobilized on Epoxy‐resin were extracted from ref. [41].

The same reactor was used to test the flow synthesis of amines. For this reaction, a high concentration of amino donor (i. e., l‐alanine) is often required (Figure 4A),^[30]^ and such excess can compete with the PLP hampering its binding to the active site of the enzyme and promoting its leaching to the reaction media under flow conditions.^[41]^ As an example, PEI‐lignin‐HeWT was utilized for the synthesis of cinnamylamine which is a valuable food additive. The influence of exogenous PLP and the flow rate on the operational stability were studied. PEI‐lignin‐HeWT reached more than 50 % conversion during 50 cycles independently on PLP supplementation (Figure S16A). Moreover, when the retention time was increased from 2 to 5 min, 90 % conversion was achieved for 50 column volumes (Figure S16B) in agreement with previous observations.^[30]^ The results obtained were compared with the performance of HeWT covalently immobilized on epoxy‐resin. Previously, our group reported a decay on the stability of immobilized HeWT during the flow synthesis of amines that could not be addressed either by adding exogenous PLP or by co‐immobilization of PLP.^[41]^ The stability of PEI‐lignin‐HeWT decreased by only 20 % (30 % when no PLP was added) after 50 column volumes (Figure 4B), whereas the same biocatalyst immobilized on epoxy resin dropped off by 50 % (70 % when no PLP was added). The high stability of the biocatalyst when immobilized on lignin may be due to its hydrophobic nature that retains the enzyme and the natural cofactor in a more efficient manner. Nevertheless, no significant improvement on STY and productivity was observed when using PEI‐lignin as the support over 50 cycles (50 min; Table S2). We believe that longer reaction times are needed to appreciate the stability effect of the PEI‐lignin on the metrics of the flow biosynthesis of cinnamylamine.

### Application of lignin for in‐line product separation

Essential to sustainable catalytic processes is the separation of the product of interest from the byproducts as well as recycling of co‐substrates. The hydrophobicity of the lignin was exploited to prepare a scavenger column connected downstream to the flow bioreactor (Table 2). In this case, an oligomeric aromatic fraction obtained as a byproduct of the hydrogenolysis of aldehyde stabilized lignin was used to add further value to the biomaterial. For comparison, a commercially available kraft lignin was also tested as a product separation column.

**Table 2:** Separation of products by a catch‐and‐release strategy from the depicted continuous flow set‐up.

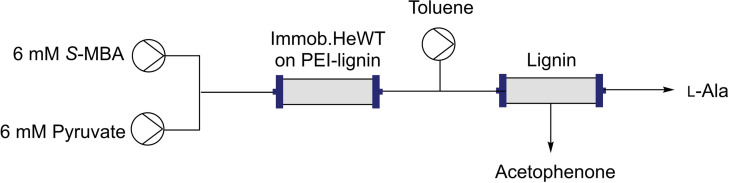				
	Trapped within column^[a]^		Desorbed with toluene^[b]^	
	Kraft lignin	Oligomer lignin	Kraft lignin	Oligomer lignin
Acetophenone	73 %	73 %	43 %	>99 %
l‐Ala	22 %	<1 %	–	–

[a] Catch step of acetophenone by hydrophobic interactions in 15 column volumes at 0.2 mL min^−1^. The percentage values refer to the initial product. [b] Purification of acetophenone by flushing with toluene for 1 h at 0.05 mL min^−1^.

As a proof of concept, the flow biocatalytic reactions were performed at 3 mm scale obtaining full conversion at *t*_R_=5 min. Afterwards, the products (acetophenone and l‐alanine) were flowed through the oligomer lignin column. The acetophenone was trapped in the lignin scavenger module due to its higher hydrophobicity while the l‐alanine was flushed out of the column. Following a catch‐and‐release strategy, the acetophenone could be recovered by adding toluene at the inlet. Overall, the performance of the oligomer lignin outperformed the kraft lignin for the separation of the products (Table 2) with 3.7 mg and 1.6 mg of acetophenone recovered, respectively. Similar values were encountered for the l‐alanine, 3.7 mg and 1.2 mg for the oligomer and the kraft lignin, respectively. These differences are likely linked to the very different features of lignin oligomers vs. kraft lignin present.^[43]^ The better performance of lignin oligomers might notably come from their lower molecular weight (*M*_w_=1940 g mol^−1^; *M*_n_=1050 g mol^−1^) compared to softwood kraft lignin (*M*_w_=5370 g mol^−1^; *M*_n_=1483 g mol^−1^), which might make product release easier compared to a more entangled matrix.

Additionally, we explored the application of the PEI‐lignin as a catch‐and‐release column for the reuse and recycling of the phosphorylated cofactor by ionic interactions. Although the enzyme (HeWT) used in this work is efficient with low amounts of PLP, the implementation of cofactor‐dependent enzymes at large scale is frequently hampered by the downstream loss of cofactors and commercial resins could help minimizing such loss.[^29^, ^44^] In this setup, PEI‐lignin was integrated as a third column in line with the PBR and the oligomer lignin for the separation of products (Figure S17). During the separation of the products, PLP, together with the l‐alanine, was flushed out of the oligomer lignin column. Then, the flow passed through the PEI‐lignin column retaining the PLP while 95 % of l‐alanine was eluted. By adding an inlet of 1 M NaCl, PLP was released achieving a recovery yield of 85 %. Finally, the cofactor solution was recirculated into the flow system and reused for another reaction obtaining similar conversion yields. This novel application of lignin may enable the integration of other PLP‐dependent enzymes on flow reactions that require exogenous addition of PLP.^[41]^ Since this strategy is based on the ionic interaction between the phosphate groups of the cofactor and the positively charged mesh of the PEI‐lignin, the versatility of the catch‐and‐release column open new paths for its implementation in the purification of other negatively charged molecules.

## Conclusion

The demand for more efficient and eco‐friendly systems is essential for the successful integration of biocatalytic reactions at industrial scale. In this work, we have exploited for first time the application of functionalized lignins and an oligomeric fraction, obtaining a flow system fully based on lignin as a matrix. We showed that this TALD‐lignin could be further modified to introduce different chemical groups, following standard procedures used for the activation of other supports. Furthermore, the advantages of lignin over other material supports for enzyme immobilization has been evidenced by the robustness and longevity of the biocatalyst in flow conditions. Nevertheless, the enzyme activity upon immobilization still needs to be improved for further implementation in industrially relevant procedures. A feasible alternative can be the combination of the TALD‐lignin with more hydrophilic materials that improve the enzyme activity upon immobilization. Additionally, both the functionalized lignin and the oligomer lignin could be shaped into microbeads for easier handling during preparation and separation from the reaction media. Finally, we envision this novel set of natural and bio‐waste lignin materials as a relevant alternative to typical immobilization supports, making biocatalytic transformations more appealing in terms of cost‐efficiency and sustainability.

## Experimental Section

### Materials

l‐Lysine hydrochloride, *S*‐methylbenzylamine, pyruvate, pyridoxal 5’‐phosphate monohydrate, ethylenediamine, 1,4‐butanediol diglycidyl ether, iminodiacetic acid, sodium borate, potassium phosphate, cobalt chloride, glucosamine, sodium periodate, l‐thiazolidine‐4‐carboxilic acid, and fluoresceine isothiocyanate were purchased from Sigma Aldrich (Gillingham, U.K). Polyethyleneimine 50 % aq. solution branched 60000 Da was acquired from Thermo Fisher Scientific. Nicotinamide adenine dinucleotide reduced form (NADH) and oxidized form (NAD^+^) were purchased from Apollo Scientific. 6‐channel μ‐slide VI was purchased from ibidi (Planegg, Germany). All other reagents were of analytical grade unless otherwise specified.

### Extraction of terephthalic aldehyde‐stabilized lignin (TALD‐lignin)

Terephthalic aldehyde stabilized lignin was extracted according to a previously published method.^[14]^ Briefly, birch wood chips (*Betula pendula*, 5 g) were introduced in a 100 mL round bottomed flask together with terephthalic aldehyde, dioxane (25 mL) and 37 wt% HCl (0.8 mL). The flask was equipped with a condenser and a magnetic stirrer and the reaction was left to proceed at 85 °C for 3 h. the mixture was then cooled to room temperature, neutralized with NaHCO_3,_ stirred for 45 min and filtered to remove the cellulose rich solid residues. The filtrate was evaporated under reduced pressure in a rotary evaporator until dry. To this, fresh dioxane (15 mL) was then added and the solution was slowly precipitated in diethyl ether (400 mL). Solid lignin was recovered after filtration and further washed with diethyl ether in a Soxhlet extraction unit for 15 h. The clean lignin was dried at 45 °C in a vacuum oven for 24 h prior to its analysis and further use (see NMR spectra in the Supporting Information).

### NMR spectroscopy of TALD‐lignin

The NMR characterization of TALD‐lignin was performed on a Bruker Avance 600 MHz spectrometer. The spectra were processed using the software Bruker TopSpin 3.6.1. ^1^H, and HSQC (Heteronuclear Single Quantum Coherence) spectra of TALD‐lignin were recorded using standard pulse sequences with some modifications: D1=10s, NS=8, P1=8us, TD=65536, O1P (F2, F1)=6.175 ppm, 125 ppm, SW (F2, F1)=13.0186 ppm, 150 ppm. The central peak of the NMR solvent was systematically used as the reference ([D_6_]DMSO *δ*_H_/*δ*_C_ 2.50/39.50).

### Diffuse‐reflectance infrared Fourier transform (DRIFT) spectroscopy of functionalized lignins.

DRIFT spectroscopy was performed on a PerkinElmer Frontier IR instrument. Lignin samples were prepared by mixing lignin (0.015 g) and potassium bromide (0.5 g) in a mortar until a homogeneous solid powder was obtained. Spectra were collected at room temperature from 500 to 4000 cm^−1^ with a scan number of 32. The background was recorded using solid KBr finely ground in a mortar.

### Production and purification of lignin oligomers

Lignin oligomers where produced by stabilization with isobutyraldehyde according to previously reported methods.[^12^, ^43^] Briefly, 1 kg of softwood, a mixture of spruce and pine previously size‐reduced in a cutting mill equipped with a 6‐mm grid, was pretreated with isobutyraldehyde (800 mL), dioxane (5.5 L) and 37 wt% HCl (250 mL) at 80 °C for 3 h. After neutralization with NaHCO_3_ (250 g), cellulose was separated from the liquor by filtration and washed with MeTHF. The liquor and the washing liquid were pooled and concentrated down under reduced pressure before being added dropwise into hexanes to precipitate lignin that was recovered as a powder after filtration and drying in a vacuum oven (45 °C).

The recovered lignin was then depolymerized in a 1 L Parr reactor. To do so, lignin (40 g), ethanol (700 mL), and 5 wt% Ni/C (20 g) were heated at 200 °C for 15 h under 20 bar H_2_. The catalyst was removed by filtration after reaction and the hydrogenolysis mixture was concentrated down under vacuum to obtain a concentrated lignin oil. Monomers were recovered through extraction of the oil with hexanes. The remaining oligomers were diluted in ethanol to decrease the viscosity until it was possible to easily transfer the mixture with a syringe. Oligomers were then precipitated in water and recovered as a powder after filtration and drying in a vacuum oven (at 45 °C). Oligomers were used in further experiments without additional treatment.

### Chemical functionalization of the lignin surface

Epoxy‐lignin (Figures 1A and S2 A): TALD‐lignin (0.5 g) was incubated with 0.3 m ethylenediamine (EDA) in 100 mm sodium bicarbonate buffer (5 mL) at pH 10 during 2 h. After washing the resin thoroughly, 1 mg mL^−1^ NaBH_4_ in the same buffer (5 mL) were added and the suspension was incubated for 1 h. After a washing step, 0.5 M 1,4‐butanediol diglycidyl ether (BDE) in 100 mm sodium bicarbonate buffer (5 mL) at pH 10 was added and the suspension was incubated for 2 h.

Epoxy‐Co^2+^‐lignin (Figures 1B and S2B): For this protocol, we used the epoxy‐lignin that we previously prepared as a starting material. Epoxy‐lignin (0.5 g) was then activated with cobalt chelate groups as described elsewhere.^[38]^ Briefly, we added modification buffer [1 mL; 0.1 m sodium borate and 2 m iminodiacetic acid (IDA) in 50 mm phosphate buffer at pH 8.0] and incubated the resin for 2 h while shaking. After filtering and washing the resin, 30 mg mL^−1^ CoCl_2_ (5 mL) was mixed with the resin for 2 h under shaking.

Co^2+^‐lignin (Figures 1C and S2 C): Epoxy‐lignin (0.5 g) was incubated with 0.5 m IDA at pH 11 overnight while shaking. After filtering and washing the resin, 30 mg mL^−1^ CoCl_2_ (5 mL) was mixed with the resin for 2 h under shaking.

PEI‐lignin (Figures 1D and S2D): TALD‐lignin (0.5 g) was incubated with 10 mg mL^−1^ polyethylenenimine (PEI) in 100 mm sodium bicarbonate buffer (5 mL) at pH 10 during 16 h (overnight). After filtering the resin, 1 mg mL^−1^ NaBH_4_ in the same buffer (5 mL) was added and the suspension was incubated for 1 h.

Glucosamine‐lignin (Figures 1E and S2E): A previous protocol for activation of acrylic carriers was adapted.^[37]^ TALD‐lignin (0.5 g) was incubated with 1.5 m glucosamine (5 mL) at pH 10 overnight. After washing the resin, 1 mg mL^−1^ NaBH_4_ in the same buffer (5 mL) was added and the suspension was incubated for 1 h. After a washing step, 15 mm NaIO_4_ (10 mL) was added and the suspension was incubated for 1 h.

After activation processes, all materials were thoroughly washed with D_2_O and kept at 4 °C until use. The presence of functional groups on the lignin was determined as described in the Supporting Information.

### Enzyme expression and purification

Gs‐Lys6DH,^[25]^ HeWT,^[28]^ He−P5C,^[29]^ and Bs‐ADH^[26]^ were expressed in *E. coli* BL21(DE3) as described in previous works. In short, for Bs‐ADH, LB media (300 mL) was inoculated with an overnight culture (3 mL) and left growing at 37 °C and 150 rpm until OD_600_ reached 0.8. At that point, the expression was induced with 1 mm IPTG and the cultures left to grow for 16 h at 30 °C and 180 rpm. For Gs‐Lys6DH and HeWT, ZYP‐5052 autoinduction media (300 mL) was inoculated with a colony transformed with the corresponding plasmid. The cultures were incubated at 37 °C and 150 rpm for 20 h. The cells were harvested by centrifugation and the pellets were resuspended in 50 mm phosphate buffer, 0.3 M NaCl, 30 mm imidazole pH 8. After sonication (5 s ON, 5 s OFF at 40 % for 6 min), the soluble fraction was separated by centrifugation, filtered with a 0.45 μm filter and purified using a Ni‐NTA column in the AKTA‐pure FPLC. The eluted protein in 50 mm phosphate buffer, 0.3 M NaCl and 300 mm imidazole pH 8 was dialyzed twice against 50 mm phosphate buffer pH 8 (containing 0.1 mm PLP in case of HeWT, and 0.1 mm DTT and 10 % glycerol for Gs‐Lys6DH).

### Enzyme immobilization procedures

All immobilization procedures were performed at 25 °C.

Immobilization on TALD‐lignin: TALD‐lignin (0.5 g) was incubated with 0.5 mg mL^−1^ enzyme solution in 100 mm sodium carbonate (5 mL) at pH 10 for 3 h while shaking. After filtering the suspension, 1 mg mL^−1^ NaBH_4_ (5 mL) was added to the resin and the suspension was incubated for 1 h while shaking.

Immobilization on epoxy‐lignin: Epoxy‐lignin (0.5 g) was incubated with 0.5 mg mL^−1^ enzyme solution in 50 mm phosphate buffer (5 mL) at pH 8 for 6 h while shaking. The remaining epoxy groups were blocked by incubation with 3 M glycine (2 mL) overnight.

Immobilization on epoxy/Co^2+^‐lignin: Epoxy‐Co^2+^‐lignin (0.5 g) was incubated with of 0.5 mg mL^−1^ enzyme solution in 50 mm phosphate buffer (5 mL) at pH 8 for 4 h while shaking. The remaining epoxy groups were blocked by incubation with 3 M glycine (2 mL) overnight.

Immobilization on PEI‐lignin: PEI‐lignin (0.5 g) was incubated with 0.5 mg mL^−1^ enzyme solution in 10 mm phosphate buffer (5 mL) at pH 8 for 1 h while shaking.

Immobilization on Co^2+^‐lignin: Co^2+^‐lignin (0.5 g) was incubated with 0.5 mg mL^−1^ enzyme solution in 50 mm phosphate buffer (5 mL) at pH 8 for 1 h while shaking.

Immobilization on glucosamine‐lignin: As the functional group exploited in the binding is an aldehyde, the procedure for immobilization follows what described above for the TALD‐lignin.

The immobilization yield was calculated as: ((activity of the free enzyme [U mg^−1^]−activity of the supernatant after immobilization [U mg^−1^])/activity of the free enzyme [U mg^−1^])×100.

### PLP co‐immobilization

The cofactor PLP was co‐immobilized following a previously reported protocol.[^41^, ^42^] For cofactor co‐immobilization on PEI‐lignin, the resin (0.5 g) was incubated with 1 mm PLP in 10 mm phosphate buffer (5 mL) at pH 8 for 1 h. The cofactor immobilization yield was determined by measuring the absorbance at 390 nm in the supernatant. For the co‐immobilization on epoxy‐lignin, a PEI‐coating step [incubation of the resin with 10 mg mL^−1^ PEI solution in 50 mm phosphate buffer (5 mL) at pH 8 for 4 h] was needed before the PLP co‐immobilization.

### Enzymatic assays

The enzymatic activities were monitored by using the microplate reader Epoch2 (Agilent).

Gs‐Lys6DH activity: Soluble enzyme (5 μL) was added to the reaction mixture (10 mm l *‐*lysine and 1 mm NAD^+^ in 200 μL of 50 mm phosphate buffer at pH 8). The increase of NADH absorbance at 340 nm was monitored for 2 min. One unit of activity was defined as the amount of enzyme that was required to reduce 1 μmol of cofactor at 25 °C.

Bs‐ADH activity: Soluble enzyme (5 μL) was added to the reaction mixture (100 mm acetone and 0.25 mm NADH in 200 μL of 25 mm phosphate buffer at pH 7). The decrease of NADH absorbance at 340 nm was monitored for 2 min. One unit of activity was defined as the amount of enzyme that was required to oxidize 1 μmol of cofactor at 25 °C.

HeWT activity: Soluble enzyme (5 μL) was added to the reaction mixture (2.5 mm *S*‐MBA, 0.25 % DMSO, 2.5 mm pyruvate and 0.01 mm PLP in 200 μL of 50 mm phosphate buffer at pH 8). The production of acetophenone was recorded by measuring the absorbance at 245 nm for 2 min. One unit of activity was defined as the amount of enzyme that was required to produce 1 μmol of acetophenone at 30 °C.

He−P5C activity: Soluble enzyme (5 μL) was added to the reaction mixture (10 mm l‐thiazolidine‐4‐carboxylic acid and 1 mm NAD^+^ in 200 μL of 50 mm phosphate buffer at pH 8). The increase of NADH absorbance at 340 nm was monitored for 2 min. One unit of activity was defined as the amount of enzyme that was required to reduce 1 μmol of cofactor at 25 °C.

Activity of immobilized enzymes on lignin: 50 mg lignin with the immobilized enzyme were mixed with the corresponding reaction mixture (5 mL) in a reaction tube with cap. The suspension was shaken at 250 rpm for 20–30 min. Samples were taken every 5 min. After centrifugation, the absorbance of 100 μL samples of the supernatant was tested.

### pH stability

50 mg of PEI‐lignin with the immobilized HeWT (5 mg g^−1^ of protein loading) were mixed with universal buffer (0.5 mL) at the corresponding pH. In case of free enzyme, a solution of 0.5 mg mL^−1^ was used. The suspension/solution was maintained for 30 min a 4 °C. At given time intervals, the residual activity was monitored as described above.

### Batch reactions and reusability

The batch reactions were performed by mixing 50 mg of lignin with the immobilized enzyme (5 mg g^−1^ of protein loading) and the reaction solution (2.5 mm pyruvate, 10 mm *S*‐MBA, and 0.1 mm PLP in 10 mm phosphate buffer at pH 8) in 2 mL microcentrifuge tubes. In the case where co‐immobilized PLP was used, no cofactor was added to the reaction mixture. The reactions were incubated at 37 °C under gentle shaking. After 2 h, 0.1 mL aliquots of the supernatant were quenched with 0.2 % HCl (0.2 mL) and acetonitrile (0.2 mL), and then analyzed by HPLC equipped with a C18 column. The compounds were detected using a UV detector at 210 nm or 250 nm after a gradient method 5 : 95 to 95 : 5 (H_2_O : MeCN 0.1 %TFA) for 4 min at 45 °C and a flow‐rate of 0.8 mL min^−1^. The retention times were 6 min for acetophenone and 4.6 min for *S*‐MBA. To test the reusability of the immobilized biocatalyst, a fresh reaction solution was added for every reaction cycle (2 h). Between each reaction cycle, the immobilized biocatalyst was separated from the reaction solution and washed with phosphate buffer.

### Microscopy studies

FITC‐labeling of enzymes: The enzymes were labeled with FITC based as described elsewhere.[^45^, ^46^] Briefly, a 1 mg mL^−1^ protein solution was mixed in a molar ratio of 1 : 20 with a FITC solution (10 mg mL^−1^ in DMSO) in 100 mm bicarbonate buffer pH 8. The mixture was incubated with gentle shaking and darkness for 1 h. Then, the unreacted FITC was removed through a 10 kDa tangential ultrafiltration unit using 100 mm bicarbonate buffer at pH 10 for Gs‐Lys6DH, and 10 mm phosphate buffer pH 8 for HeWT. Finally, the labeled enzymes were immobilized on lignin following the protocols described above.

Fluorescence microscopy imaging: The suspension of immobilized enzymes on lignin was filtered and placed on a 8‐well μ‐slide. Glycerol (200 μL) was added to improve the match in refractive index between the medium and the opaque lignin. The images were taken using a Nikon Ti2 Eclipse confocal microscope with a X‐light V2 spinning disk. Objectives 20x (air) and 40x (oil) were used. FITC‐labeled enzymes were observed using *λ*_ex_=470 nm and the emission filter 515 nm. The autofluorescence of PLP was visualized using *λ*_ex_=395 nm and the emission filter 432 nm.

### Continuous flow biocatalytic reactions

Flow biotransformations were performed by using R2S/R4 vapourtec flow reactor equipped with a V3 pump and an Omnifit glass column (6.6 mm i.d.×100 mm length) filled with the immobilized HeWT (5 mg g^−1^) on PEI‐lignin. For the deamination reactions, a solution of amino donor was mixed with the amino acceptor solution in a T‐tube resulting into 5 mm pyruvate, 5 mm *S*‐MBA, and 0.1 mm PLP in 10 mm phosphate buffer pH 8 containing 2.5 % DMSO. For the synthesis of cinnamylamine, the resulting concentrations after the T‐tube were 5 mm trans‐cinnamaldehyde, 250 mm l‐alanine, and 0.1 mm PLP in 10 mm phosphate buffer pH 8 containing 2.5 % DMSO. In both reactions, the flow stream was driven to the PBR (reactor volume: 0.95 mL, temperature: 37 °C, pressure: 0.3–0.4 bars). A first equilibration step was performed for 60 min at 0.25 mL min^−1^. Then, the flow rate was maintained at 0.19 mL min^−1^ with a residence time of 5 min. The products were analyzed by HPLC as described above. Cinnamaldehyde (*t*_R_=6.1 min) was monitored at 210 nm, and cinnamylamine (*t*_R_=4.8 min) at 250 nm.

### In‐flow product separation

The product from the deamination flow reaction (at 3 mm scale) was directed to a catch‐and‐release column with oligomer/kraft lignin (column volume: 2.3 mL, pressure: 0.1 bars) which retained the acetophenone into the column. Afterwards, toluene (0.65 mL min^−1^) was added at the inlet to desorb and recover the acetophenone. Acetophenone (*t*_R_=6 min) and l‐alanine (FMOC derivatization, 210 nm, *t*_R_=6.3 min) were monitored by HPLC.

## Conflict of Interest

S.B. and J.S.L. are inventors on a European patent application (EP19202957) submitted by EPFL and that covers the isolation of functionalized lignins via the aldehyde assisted process. JSL is co‐founder and part owner, and JBB part owner of Bloom Biorenewables Ltd that aims at commercializing the aldehyde assisted fractionation process.

## Supporting information

As a service to our authors and readers, this journal provides supporting information supplied by the authors. Such materials are peer reviewed and may be re‐organized for online delivery, but are not copy‐edited or typeset. Technical support issues arising from supporting information (other than missing files) should be addressed to the authors.

Supporting Information
