# Supplementary material for: Dual Valorization of Lignin as a Versatile and Renewable Matrix for Enzyme Immobilization and (Flow) Bioprocess Engineering
Source: ChemSusChem. 2021 Jul 5;14(15):3198–207. doi: 10.1002/cssc.202100926 (PMC8457236; doi:10.1002/cssc.202100926)
Supplement: Supplementary file 1 — Supporting Information [file CSSC-14-3198-s001.pdf]

ChemSusChem

Supporting Information

Dual Valorization of Lignin as a Versatile and Renewable Matrix for Enzyme Immobilization and (Flow) Bioprocess Engineering

Ana I. Benítez-Mateos, Stefania Bertella, Jean Behaghel de Bueren, Jeremy S. Luterbacher,* and Francesca Paradisi*© 2021 The Authors. ChemSusChem published by Wiley-VCH GmbH. This is an open access article under the terms of the Creative Commons Attribution License, which permits use, distribution and reproduction in any medium, provided the original work is properly cited.

Content

Figure S1. HSQC-NMR and FTIR analysis of lignins. 2

Figure S2. Schemes of the functionalization procedures of lignin 3

Determination of the functional groups on the lignin 3

Figure S3. Analysis of the immobilization of enzymes on lignin. 4

Figure S4. Fluorescence microscopy images of enzymes immobilized on lignin. 5

Figure S5. SDS-PAGE (12%) of the immobilization of Gs-Lys6DH on reduced aldehyde-lignin. 5

Figure S6. Effect of the degree of aldehyde activation of lignin on the recovered enzyme activity. 6

Figure S7. Immobilization of enzymes on lignin in presence of additives to reduce the hydrophobic interactions. 6

Figure S8. Immobilization of Gs-Lys6DH on glucosamine-lignin. 7

Figure S9. SDS-PAGE of the immobilization, stability and recycling of HeWT on PEI-lignin. 7

Figure S10. Optimization of the protein loading capacity of PEI-lignin. 8

Figure S11. Stability of HeWT immobilized on PEI-lignin against solvents and pH. 8

Figure S12. Co-immobilized HeWT and PLP on PEI-lignin. 9

Figure S13. SDS-PAGE of the stability of immobilized HeWT during flow reactions. 10

Table S1. Complementary metrics for the evaluation of the flow reactors depicted in Figure 3. 10

Figure S14. Influence of exogenous PLP on the stability of immobilized HeWT on PEI-lignin during flow deamination reactions. 11

Figure S15. Stability of the biocatalyst at 1 min of r.t. during flow deamination reactions. 11

Figure S16. Stability of HeWT immobilized on PEI-lignin during the synthesis of cinnamylamine. 12

Table S2. Complementary metrics for the evaluation of the flow reactors depicted in Figure 4. 12

Figure S17. Flow set-up with catch-and-release step of PLP by ionic interactions. 13

A

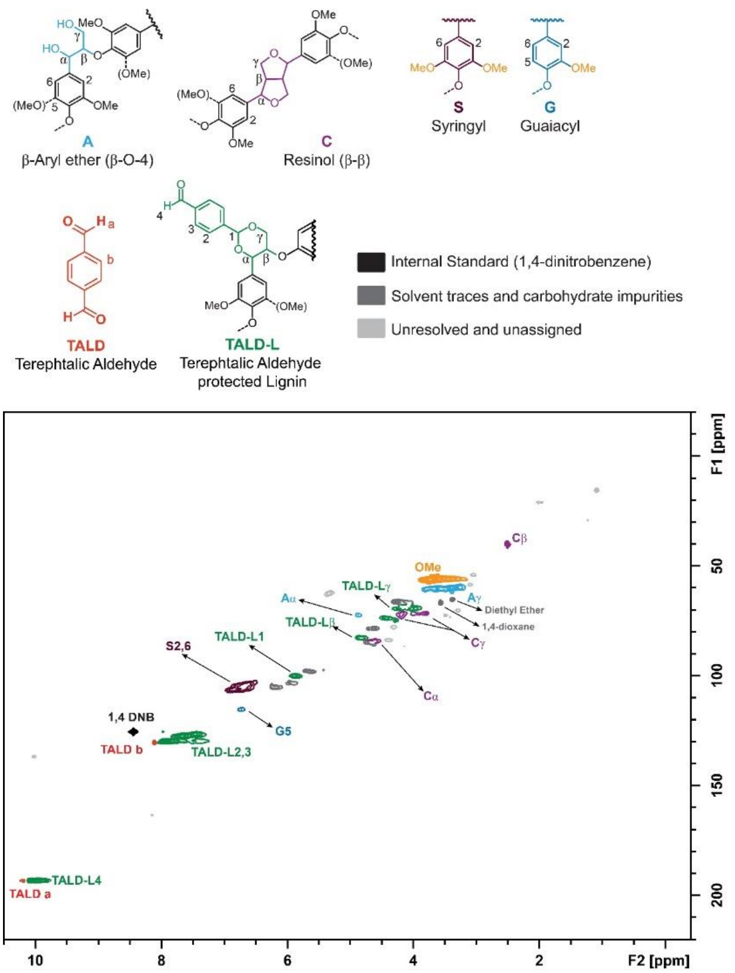

B

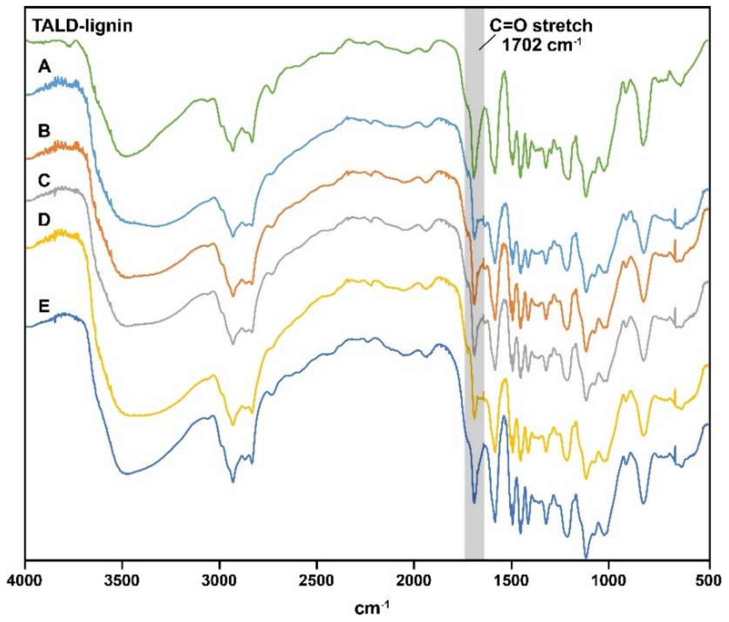

Figure S1. HSQC-NMR and DRIFT analysis. A) HSQC-NMR of TALD-lignin. B) DRIFT spectra of TALD-linin and functionalized lignins. A: Epoxy-lignin, B: Epoxy/Co^{2+}-lignin, C: PEI-lignin, D: Co^{2+}-lignin, E: glucosamine-lignin.

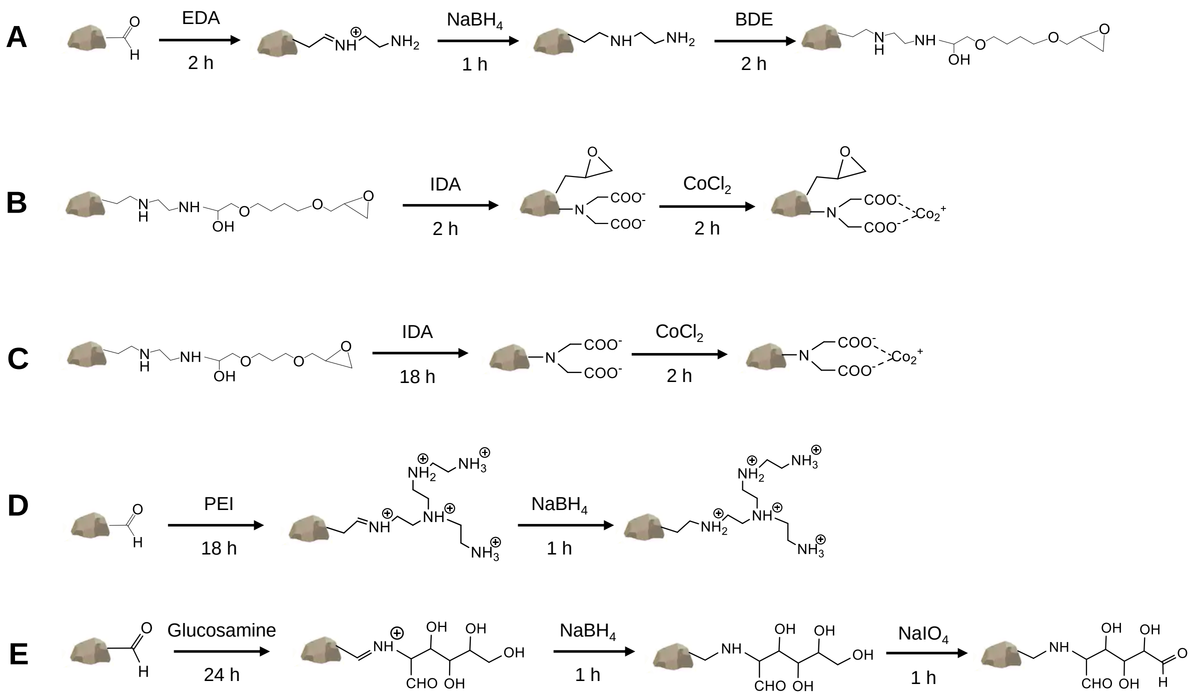

Figure S2. Schemes of the functionalization procedures of lignin. A) Activation of PEI-lignin. B) Activation of Epoxy-lignin. C) Activation of Epoxy/Co^{2+}-lignin. D) Activation of Co^{2+}-lignin. E) Activation of Glucosamine-lignin.

Assessment of the functionalization of lignin

- <u>Quantification of epoxy groups:</u> A previous reported protocol was adapted to lignin and followed.[1] 50 mg of epoxy-lignin, epoxy-Co^{2+}-lignin, or Co^{2+}-lignin were mixed with 0.5 mL of 0.5 M H_2SO_4 and incubate for at 1 hour under gentle agitation. Then, the lignin was filtered and washed thoroughly with $H_2$0 (at least 10 times with 10 volumes of $H_2$0). 0.5 mL of 10 mM $NaIO_4$ were added to the lignin and the suspension was incubated for 1 h under gentle agitation. After filtering the lignin, 0.1 mL of the supernatant were taken and mixed with 0.5 mL of saturated bicarbonate and 0.5 mL of 10% KI. 0.1 mL of the previous solution was withdrawn and placed on a 96-well plate. The absorbance was measured at 405 nm. KI reacts quickly with $NaIO_4$ and I^- is formed giving a yellow colour. TALD-lignin was used as a control, and no colour formation was observed. The following number of epoxy groups were determined:

Epoxy-lignin	Epoxy-Co^{2+}-lignin	Co^{2+}-lignin
129 μmol/g	45 μmol/g	0 μmol/g

- <u>Quantification of aldehyde groups on glucosamine-lignin</u>: A previous reported protocol was adapted to lignin and followed.[1,2] After the incubation step with $NaIO_4$, 0.5 mL of the supernatant were taken and the same protocol for quantification of epoxy groups was followed by adding saturated bicarbonate and KI. As result, full activation with glucosamine was determined.

- Assessment of amino groups: A previous reported protocol was adapted to lignin and followed.[3] 50 mg of PEI-lignin were added to 50 μL de 2,4,6-trinitrobenzenesulfonic acid (TNBSA) and 500 μL of 100 mM sodium bicarbonate buffer at pH 10. The suspension was incubated under gentle agitation for 10 min. After centrifugation, the supernatant was removed, and the lignin was washed with 3 M NaCl and a final wash with H_2O. TNBSA reacts with primary amines forming a yellow-orange product.

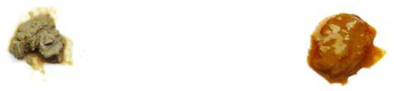

TALD-lignin PEI-lignin

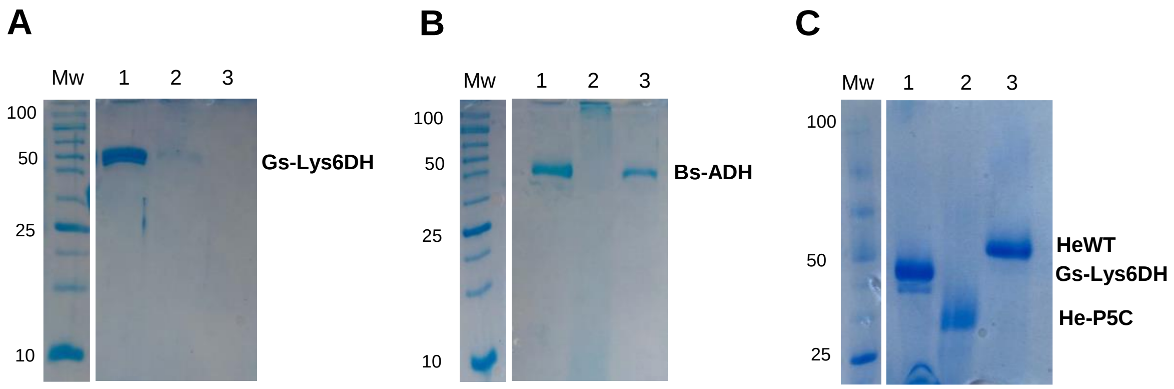

Figure S3. Analysis of the immobilization of enzymes on lignin. A) 14% SDS-PAGE. Line 1: free Gs-Lys6DH. Line 2: immobilized Gs-Lys6DH on TALD-lignin. Line 3: supernatant after the immobilization of Gs-Lys6DH on TALD-lignin. B) 14% SDS-PAGE. Line 1: free Bs-ADH. Line 2: immobilized Gs-ADH on TALD-lignin. Line 3: supernatant after the immobilization of Gs-ADH on TALD-lignin. C) 12% SDS-PAGE. Line 1: immobilized HeWT on Co^{2+}-lignin. Line 2: immobilized He-P5C on Co^{2+}-lignin. Line 3: immobilized HeWT on Co^{2+}-lignin. Mw: Broad range protein marker from NEB.

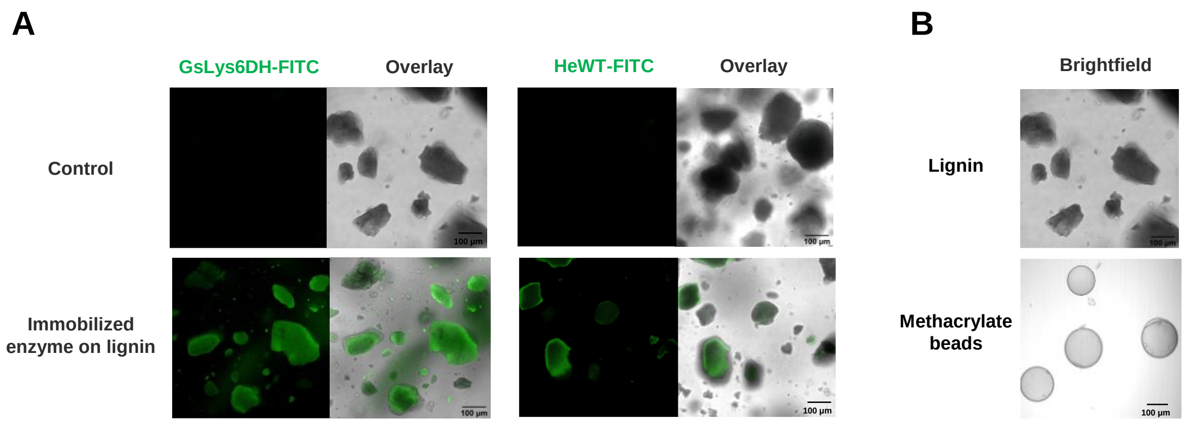

Figure S4. Fluorescence microscopy images of enzymes immobilized on lignin. A) Left column, Gs-Lys6DH labelled with FITC and immobilized on TALD-lignin. Right column, HeWT labelled with FITC and immobilized on PEI-lignin. Control samples refer to the lignin without immobilized enzymes. The images were corrected applying the same filter to compensate the background fluorescence level. B) Transmission images comparing lignin and methacrylate microbeads.

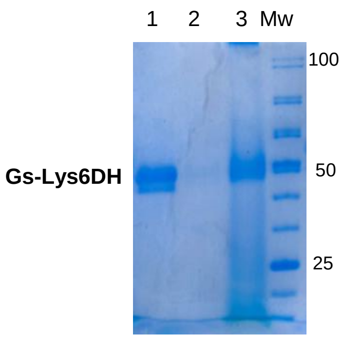

Figure S5. SDS-PAGE (12%) of the immobilization of Gs-Lys6DH on reduced TALD-lignin. Line 1: free Gs-Lys6DH. Line 2: supernatant after the immobilization of Gs-Lys6DH on reduced TALD-lignin. Line 3: immobilized Gs-Lys6DH on reduced aldehyde-lignin.

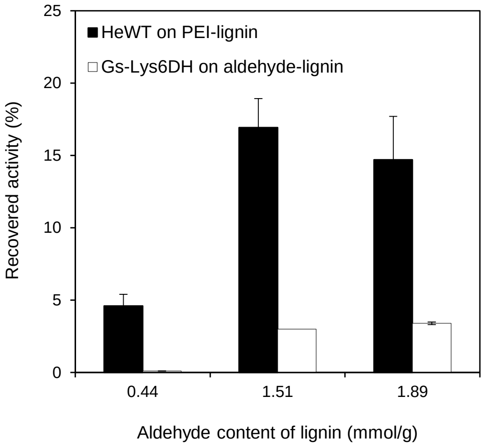

Figure S6. Effect of the degree of aldehyde activation of lignin on the recovered enzyme activity. The offered enzyme was 5 mg/g. In all cases, the immobilization yield reached >99%.

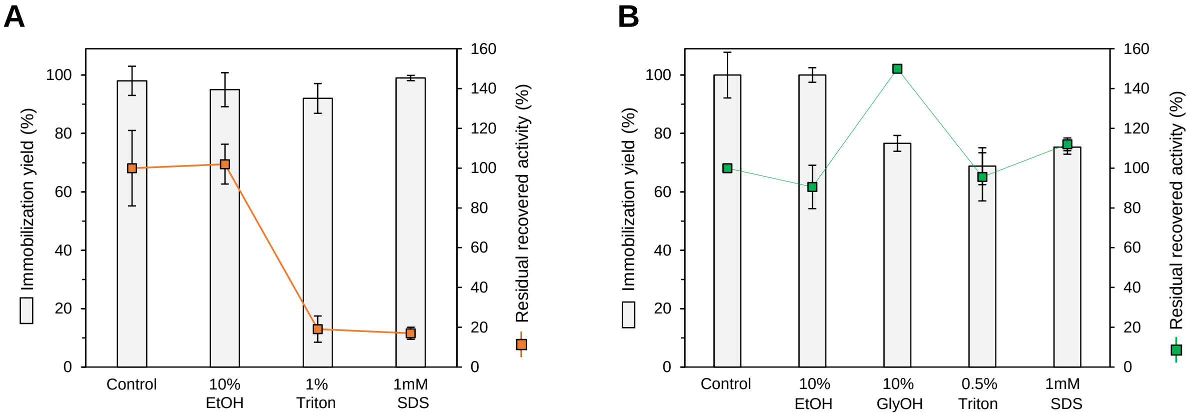

U/g	0.1	0.1	<0.1	<0.1

U/g	1.6	1.5	1.5	1.1	1.2

Figure S7. Immobilization of enzymes on lignin in presence of additives to reduce the hydrophobic interactions. A) Gs-Lys6DH immobilized on aldehyde-lignin. B) HeWT immobilized on PEI-lignin. In all cases, the offered enzyme was 5mg/g. To determine the residual recovered activity, the recovered activity of the immobilized enzyme was compared with the activity of the free enzyme in presence of the same additive during the same incubation time.

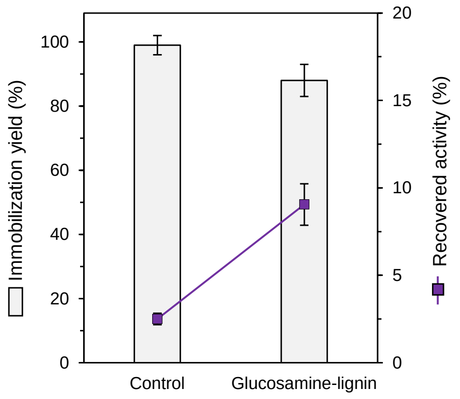

U/g	0.1	0.2

Figure S8. Immobilization of Gs-Lys6DH on glucosamine-lignin. Control sample refers to the enzyme immobilized on aldehyde-lignin. The offered enzyme was 5 mg/g.

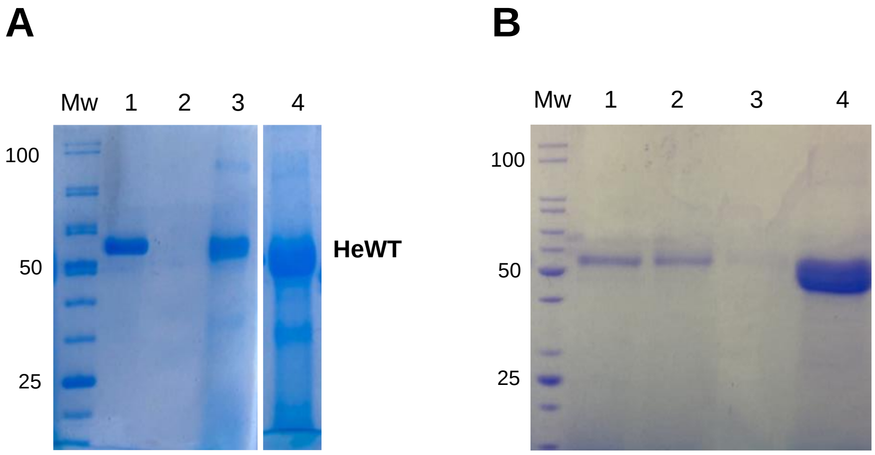

Figure S9. SDS-PAGE of the immobilization, stability and recycling of HeWT on PEI-lignin. A) Line 1: free HeWT. Line 2: supernatant after immobilization of HeWT on PEI-lignin. Line 3: HeWT immobilized on PEI-lignin. Line 4: HeWT immobilized on PEI-lignin after 8 batch reactions. B) Line 1: after incubation with 3 M NaCl for 30 min. Line 2: after incubation with ethanol for 30 min. Line 3: after sequential incubation with 3 M NaCl for 30 min, and ethanol for 30 min. Line 4: Recycle of lignin to re-immobilize HeWT at a high protein loading (50 mg/g). Mw: Broad range protein marker from NEB.

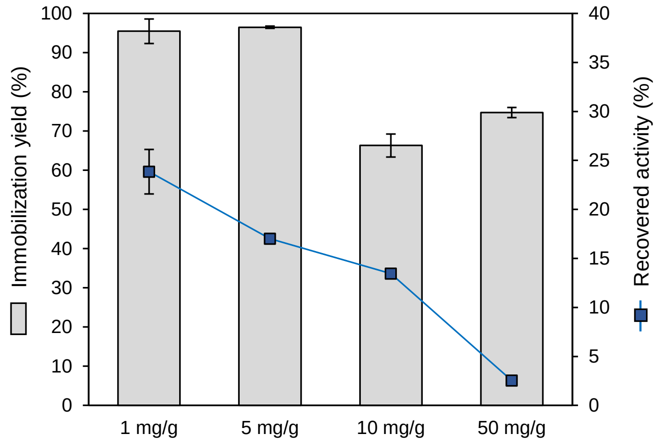

U/g	0.5	1.8	1.9	1.9

Figure S10. Optimization of the protein loading capacity of PEI-lignin. U/g corresponds to the expressed activity of immobilized HeWT.

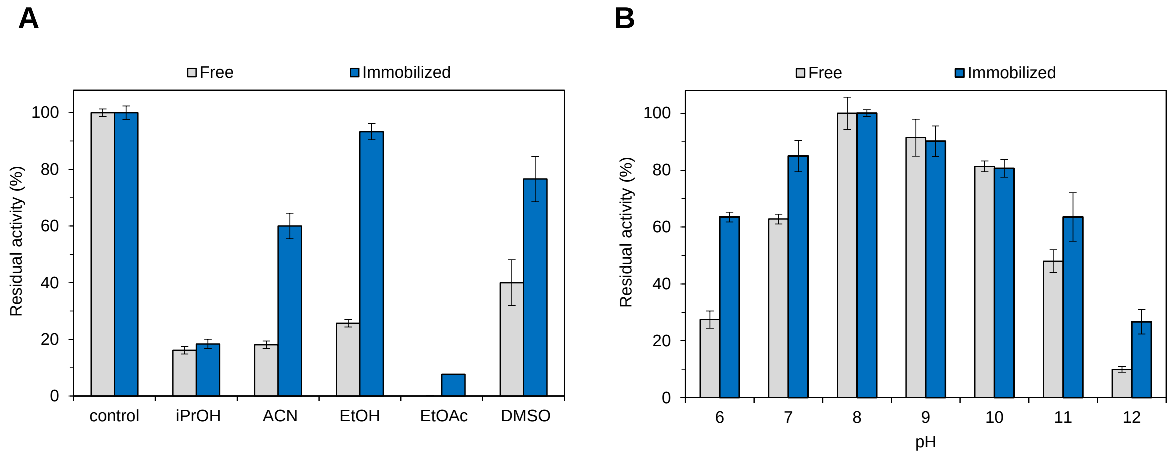

Figure S11. Stability of HeWT immobilized on PEI-lignin against solvents and pH. A) Activity of HeWT in presence of 20% of different cosolvents. B) Activity of HeWT after incubation at different pH for 30 min at 4ºC.

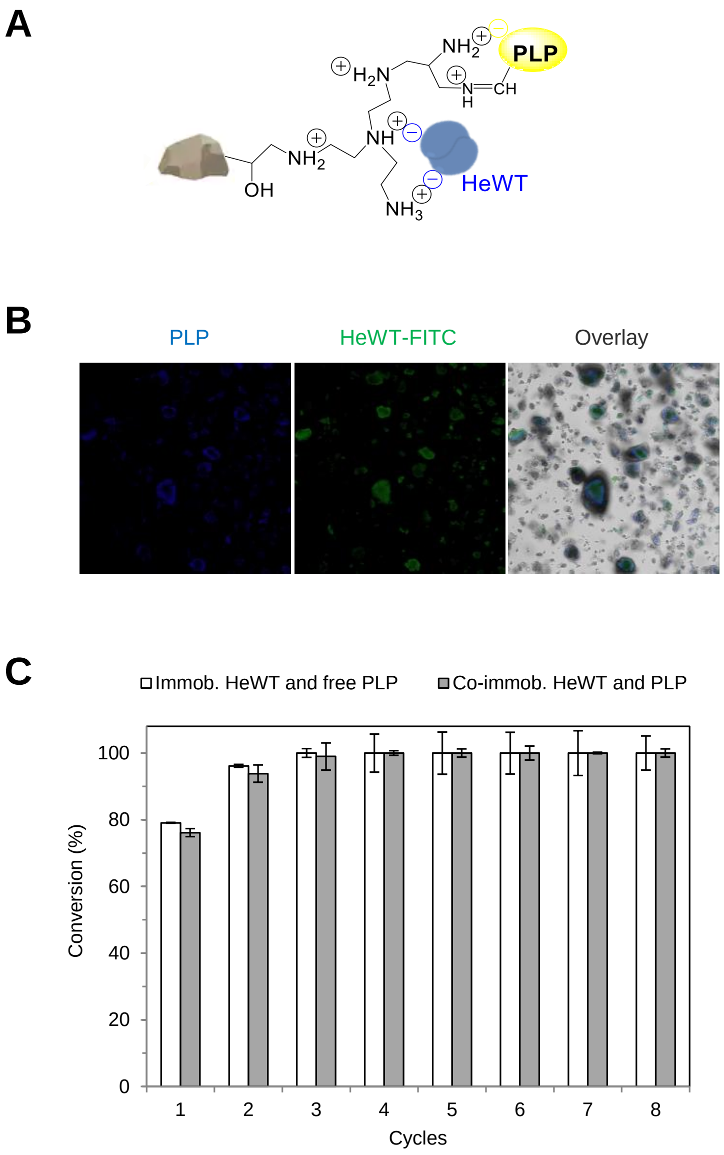

Figure S12. Co-immobilized HeWT and PLP on PEI-lignin. A) Scheme of the co-immobilization of HeWT (ionic interaction) and PLP (ionic interactions and imine bonds). B) CLSM imaging the co-immobilized HeWT (labeled with FITC, in green) and PLP (autofluorescent, in blue). The overlay shows the merge of the fluorescence and the transmission images. Due to the high autofluorescence of lignin, the images were corrected applying the same filter to compensate the background. C) Batch reactions and reuses were performed at 37 ºC with a reaction mix containing 2.5 mM pyruvate, 2.5 mM S-MBA and 0.1 mM PLP (when the PLP was co-immobilized, no free PLP was added) in 10 mM phosphate buffer at pH 8. Each cycle corresponds to 2 hours.

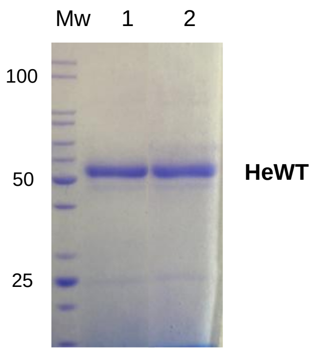

Figure S13. SDS-PAGE of the stability of immobilized HeWT during flow reactions. Line 1: HeWT immobilized on PEI-lignin before the flow reactions. Line 2: HeWT immobilized on PEI-lignin after flow reactions. Mw: Broad range protein marker from NEB.

Table S1. Complementary metrics for the evaluation of the flow reactors depicted in Figure 3. The space-time yield (STY) was calculated [a]in the first column volume and [b]after 40 column volumes (3.3 h). The total productivity and the turnover number (TN) within 40 column volumes were calculated.

Support	Protein loading (mg/g)	Activity on the lignin (U/g)	STY (g/L/h)		Productivity ($mg_{product}/mg_{enzyme}/h$)	TN ($\mu mol_{product}/min/mg_{enzyme}$)
			[a] Initial	[b] Final		
Pu-Co^{2+}/eA	1	0.78	14.42	2.6	9.45	1.31
EC-Co^{2+}/eA	1	0.84	14.42	3.71	9.7	1.34
PEI-lignin	1	0.5	8.6	8.15	8.35	1.16

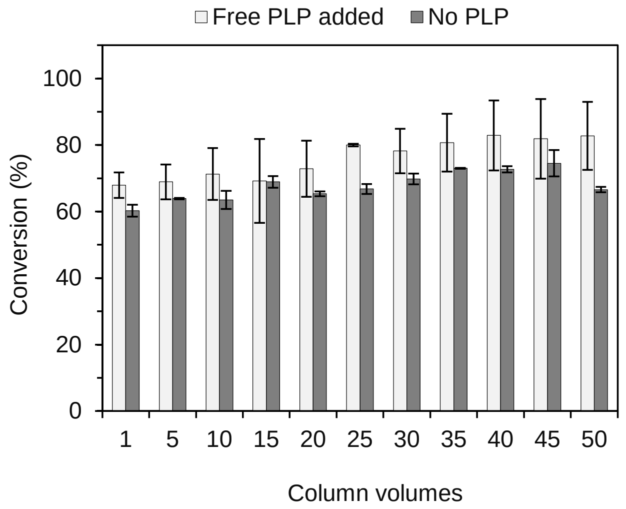

Figure S14. Influence of exogenous PLP on the stability of immobilized HeWT on PEI-lignin during flow deamination reactions. In case of exogenous free PLP, 0.1 mM was added. Each column volume corresponds to 5 min.

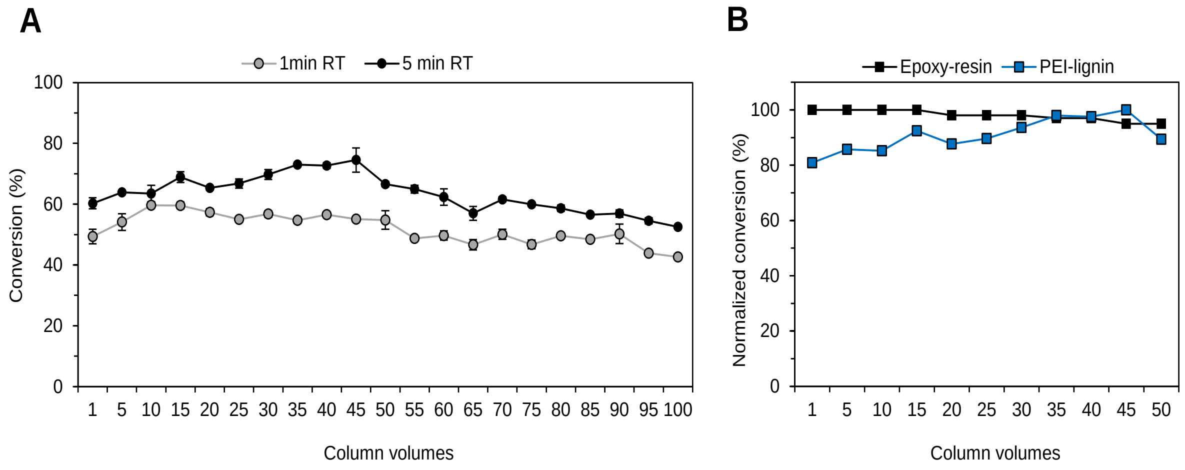

Figure S15. Stability of the biocatalyst at 1 min of r.t. during flow deamination reactions. No PLP was added to the reaction mix. A) HeWT immobilized on PEI-lignin. The flow rates were 0.2 mL/min (5 min R.T.) and 0.5 mL/min (1 min R.T.). B) HeWT immobilized on different supports by covalent bonds (epoxy-resin) and reversible interactions (PEI-lignin). Each column volume corresponds to 1 min.

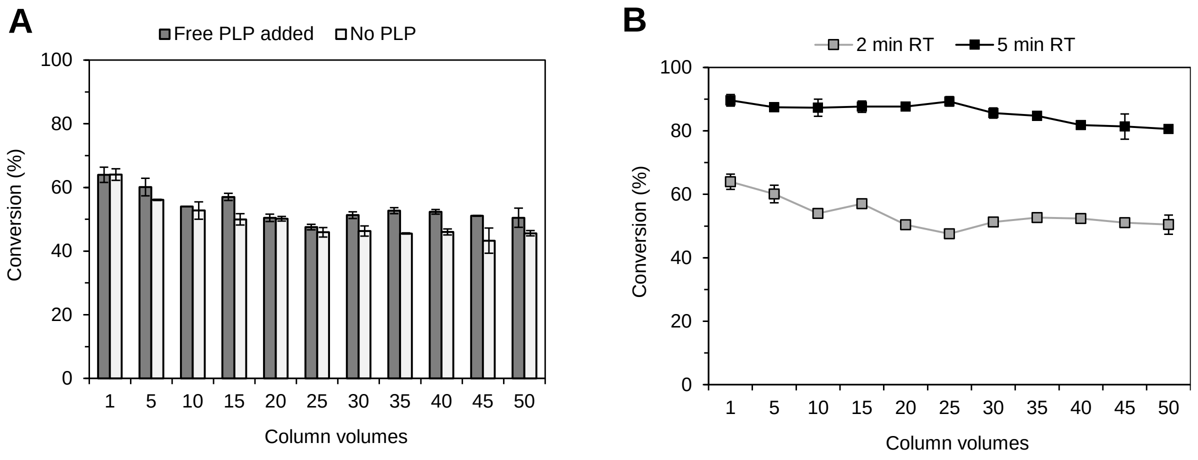

Figure S16. Stability of HeWT immobilized on PEI-lignin during the synthesis of Cinnamylamine. A) Influence of exogenously added PLP. Each column volume corresponds to 2 min. B) Effect of the flow rate on the productivity of cinnamylamine. 0.1 mM PLP was added.

Table S2. Complementary metrics for the evaluation of the flow reactors depicted in Figure 4. The space-time yield (STY) was calculated [a]in the first column volume and [b]after 50 column volumes (50 min). The total productivity and the turnover number (TN) within 50 column volumes were calculated.

Support	Protein loading (mg/g)	Activity on the lignin (U/g)	STY (g/L/h)		Productivity ($mg_{product}/mg_{enzyme}/h$)	TN ($\mu mol_{product}/min/mg_{enzyme}$)
			[a] Initial	[b] Final		
Epoxy-resin + free PLP	5	3.2	39.96	20.81	4.66	0.58
Epoxy-resin	5	3.2	39.96	11.99	4.2	0.53
PEI-lignin + free PLP	5	1.8	25.57	20.17	4.3	0.54
PEI-lignin	5	1.8	25.60	18.22	3.98	0.50

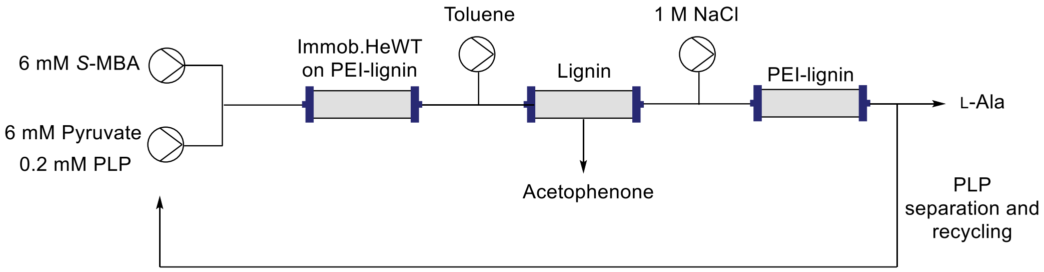

Figure S17. Flow set-up with catch-and-release step of PLP by ionic interactions. Flow-rate: 0.2 mL/min.

Supporting References:

(1) Guisán, J. M. Aldehyde-Agarose Gels as Activated Supports for Immobilization-Stabilization of Enzymes. *Enzyme Microb. Technol.* **1988**, *10* (6), 375–382. https://doi.org/10.1016/0141-0229(88)90018-X.

(2) Serra, I.; Benucci, I.; Robescu, M. S.; Lombardelli, C.; Esti, M.; Calvio, C.; Pregnolato, M.; Terreni, M.; Bavaro, T. Developing a Novel Enzyme Immobilization Process by Activation of Epoxy Carriers with Glucosamine for Pharmaceutical and Food Applications. *Catalysts* **2019**, *9* (10), 843. https://doi.org/10.3390/catal9100843.

(3) Snyder, S. L.; Sobocinski, P. Z. An Improved 2,4,6-Trinitrobenzenesulfonic Acid Method for the Determination of Amines. *Anal. Biochem.* **1975**, *64* (1), 284–288. https://doi.org/10.1016/0003-2697(75)90431-5.
